# Priority accuracy by dispatch centers and Emergency Medical Services professionals in trauma patients: a cohort study

**DOI:** 10.1007/s00068-021-01685-1

**Published:** 2021-05-21

**Authors:** Job F. Waalwijk, Robin D. Lokerman, Rogier van der Sluijs, Audrey A. A. Fiddelers, Luke P. H. Leenen, Mark van Heijl, Martijn Poeze, the Pre-hospital Trauma Triage Research Collaborative (PTTRC), Koen W. W. Lansink, Mariska A. C. de Jongh, Dennis den Hartog, Jens A. Halm, Georgios F. Giannakópoulos, Michael J. R. Edwards, Pierre M. van Grunsven, Wim Breeman, Risco van Vliet, Thijs F. Verhagen, Margreet W. M. J. Hoogeveen, Leontien M. Sturms

**Affiliations:** 1 Department of Surgery, Maastricht University Medical Center, Maastricht, The Netherlands; 2 Department of Surgery, University Medical Center Utrecht, Utrecht, The Netherlands; 3 Network Acute Care Limburg, Maastricht University Medical Center, Maastricht, The Netherlands; 4 Center for Artificial Intelligence in Medicine and Imaging, Stanford, USA; 5 Department of Surgery, Diakonessenhuis Utrecht/Zeist/Doorn, Utrecht, The Netherlands

**Keywords:** Dispatch priority, Emergency Medical Services, Accuracy, Field triage, Trauma

## Abstract

**Purpose:**

Priority-setting by dispatch centers and Emergency Medical Services professionals has a major impact on pre-hospital triage and times of trauma patients. Patients requiring specialized care benefit from expedited transport to higher-level trauma centers, while transportation of these patients to lower-level trauma centers is associated with higher mortality rates. This study aims to evaluate the accuracy of priority-setting by dispatch centers and Emergency Medical Services professionals.

**Methods:**

This observational study included trauma patients transported from the scene of injury to a trauma center. Priority-setting was evaluated in terms of the proportion of patients requiring specialized trauma care assigned with the highest priority (i.e., sensitivity), undertriage, and overtriage. Patients in need of specialized care were defined by a composite resource-based endpoint. An Injury Severity Score ≥ 16 served as a secondary reference standard.

**Results:**

Between January 2015 and December 2017, records of 114,459 trauma patients were collected, of which 3327 (2.9%) patients were in need of specialized care according to the primary reference standard. Dispatch centers and Emergency Medical Services professionals assigned 83.8% and 74.5% of these patients with the highest priority, respectively. Undertriage rates ranged between 22.7 and 65.5% in the different prioritization subgroups. There were differences between dispatch and transport priorities in 17.7% of the patients.

**Conclusion:**

The majority of patients that required specialized care were assigned with the highest priority by the dispatch centers and Emergency Medical Services professionals. Highly accurate priority criteria could improve the quality of pre-hospital triage.

**Supplementary Information:**

The online version contains supplementary material available at 10.1007/s00068-021-01685-1.

## Introduction

A fundamental element of modern trauma systems is to transport the right patient to an appropriate hospital. The transportation of patients in need of specialized care to lower-level trauma centers (i.e., undertriage) is associated with increased mortality and morbidity [1–4]. Overtriage—transporting patients without the need for specialized care to higher-level trauma centers—leads to unnecessary use of limited resources and increasing costs [5]. In addition, trauma patients requiring specialized care are likely to benefit from expedited examination and treatment by Emergency Medical Services (EMS) professionals and swift transportation to a higher-level trauma center [6].

Dispatch priority and the priority assigned by EMS professionals (i.e., transport priority) are major factors that affect response and transport times of ambulances. Therefore, accurate prioritization is of great importance to achieve optimal patient outcomes. The dispatch operator—receiving the initial emergency call—determines whether the patient requires assessment by an EMS professional and sets the priority of the emergency request. After assessment of injury severity on-scene, EMS professionals reconsider the priority. Subsequently, they can decide to set a higher or lower priority or to maintain the priority as assigned by the dispatch center. Minimizing discrepancies between dispatch and transport priority can optimize resource utilization in the pre-hospital setting. Different emergency medical dispatch protocols have been developed to achieve optimal prioritization of ambulances. The accuracy of dispatch center protocols in identifying time critical conditions, including but not limited to trauma patients, was reported to range between 78 and 93% [7]. To our knowledge, the accuracy of transport priority has not been investigated before.

The current study was designed to evaluate the accuracy of dispatch center and EMS professional prioritization of trauma patients in need of specialized care. Furthermore, it was our aim to determine undertriage and overtriage rates and to quantify the differences between dispatch and transport priorities.

## Methods

### Study design and setting

This multisite, cohort study aimed to evaluate the accuracy of priority-setting by dispatch centers and EMS professionals. Five different EMS regions (Brabant Midden-West, Brabant-Noord, Gelderland-Zuid, Rotterdam-Rijnmond, Utrecht) and seven trauma regions in the Netherlands participated in the current study. These EMS regions transport nearly 380,000 patients annually, covering an area of approximately 6700 square kilometers with 4.7 million inhabitants. Dispatch centers professionals are nurses with additional training for dispatch [8]. The ambulances are staffed by a nurse that is licensed to provide advanced life support care and a driver that is qualified to provide medical assistance. In the Netherlands, three levels of priority exist: (i) A1 is the highest priority and implies an acute threat to the patients’ vital functions only to be excluded after an on-scene evaluation by the EMS professional, (ii) A2 priority indicates a request for care without a direct threat to life, but may involve serious damage to the patients’ health, and (iii) low-priority are scheduled transports (e.g., inter-facility transfers) [8, 9]. The participating trauma regions contain seven higher-level trauma centers and 60 lower-level trauma centers. All hospitals in these regions include a trauma-receiving emergency department and only level I trauma centers (i.e., higher-level trauma centers) are designated to treat patients in need of specialized care. Level II and level III trauma centers (i.e., lower-level trauma centers) are capable to provide care to mildly and moderately injured patients. This study adhered to the Strengthening the Reporting of Observational Studies in Epidemiology guidelines [10]. The Medical Ethical Committee of the University Medical Center Utrecht judged this study as not subject to the Medical Research Involving Human Subjects Act (reference number 20/500747).

### Selection of participants

**Fig. 1:**
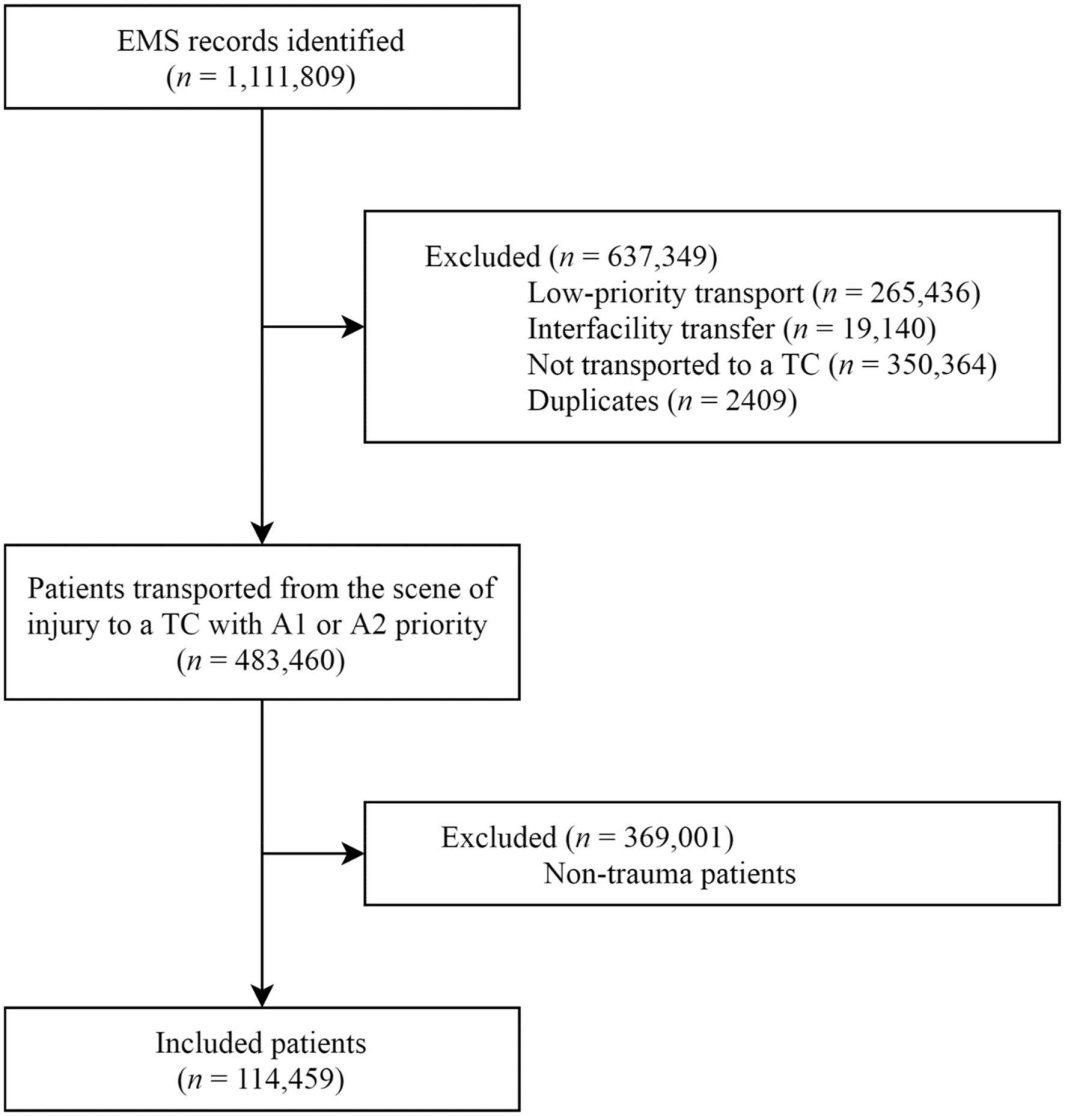
Patient inclusion flowchart. *EMS* Emergency Medical Services. *TC* Trauma Center

All trauma patients assigned with A1 or A2 priority by the dispatch center that were transported by ground ambulance to a trauma center were included. Low-priority transports, inter-facility transfers, patients that were not transported to a participating trauma center (e.g., treatment by EMS professional on-site), duplicates, and non-trauma patients were excluded. The patient inclusion strategy is depicted in Fig. 1. A selection tool that was developed in prior research was used to automatically identify trauma patients in unfiltered EMS records [11]. This tool demonstrated an accuracy of 98.9% (95% CI, 98.3–99.2) through external validation.

### Data collection

Ambulance records from the participating EMS regions were prospectively collected from January 1, 2015 through December 31, 2017. These records consisted of dispatch and transport priority, patient demographics, injury locations, pre-hospital time intervals, vital signs, and the receiving hospital. The vital signs included systolic blood pressure, respiratory rate, and the Glasgow Coma Scale. Patient locations were converted to coordinates and the driving distance to destinations was calculated using Bing Maps, accounting for day of the week and hour of the day [12]. Response time was defined as the time between ambulance dispatch and arrival at the scene of injury, whereas transport time covers the time between departure from the scene of injury and arrival at the trauma center. The ambulance records were matched with data from regional trauma registries. These registries contain, among others, diagnosed injuries, ISSs, and mortality status of all admitted trauma patients. Patients that were discharged from the emergency department were assumed not to be in need of specialized care, nor to be severely injured, as verified in data from prior research [13]. EMS and hospital records were linked with unique EMS identifier. Patients with a missing identifier in either pre-hospital or in-hospital data were linked using a prediction model from prior research that demonstrated an accuracy of 100.0% [11]. Several variables, such as date of injury and transportation destination, are incorporated in this model.

### Outcomes and definitions

The main outcome of this study was the sensitivity of dispatch and transport priority, defined as the proportion of patients requiring specialized trauma care assigned with the highest priority. Patients were classified as being in need of specialized care when they were (i) admitted to the intensive care unit after initial evaluation in the emergency department, (ii) underwent an emergency intervention within 24 h, (iii) died within 24 h after arrival at the trauma center, or (iv) when they were intubated pre-hospital (Table 1). This composite resource-based endpoint (i.e., early critical-resource use) is comparable to previously suggested definitions [11, 14–16]. An Injury Severity Score (ISS) ≥ 16 served as a secondary reference standard for being in need of specialized care [17]. Secondary outcomes were the undertriage and overtriage rates in different priority subgroups. The proportion of patients requiring specialized care, according to both reference standards, initially transported to a lower-level trauma center was considered undertriage. Overtriage was defined as the proportion of patients not in need of specialized care that were transported to a higher-level trauma center.

**Table 1:** Definition of resource-based reference standard (early critical-resource use)

Admittance to intensive care unit after initial evaluation in emergency department
Emergency intervention within 24 h
Damage control thoracotomy
Damage control laparotomy
Damage control orthopedics
Extra peritoneal pelvic packing
Revascularization of extremities
Craniotomy
Intracranial pressure monitoring
Coniotomy/cricothyrotomy
Interventional radiology
Death within 24 h after arrival at trauma center
Pre-hospital intubation
Patients that met one of these criteria were classified as being in need of specialized care

### Data analysis

Data were analyzed using R statistical software (R version 3.6.1) [18]. Multilevel multiple imputation was used to account for missing values across different sites using the package *micemd* [19]. Variables with missing values were dispatch priority (0.1%), transport priority (3.0%), systolic blood pressure (29.1%), respiratory rate (36.6%), Glasgow Coma Scale (13.6%), response time (1.4%), and transport time (4.1%). The predictor matrix that was used to impute these missing values included, among others, age, gender, vital parameters measured in the emergency department, and in-hospital outcomes. Forty-eight datasets were generated based on 20 iterations per set. Quantitative variables were described using median and interquartile ranges, or the mean and its corresponding standard deviation. Frequencies and proportions were computed for nominal variables. Groups were compared with the Mann–Whitney *U* or *χ*^2^ test. *p* values below 0.05 were considered statistically significant. Analyses were applied to each of the 48 datasets. Point estimates of sensitivity, undertriage, and overtriage were calculated with the average results of all analyses and Agresti–Coull confidence intervals were computed, in accordance with Rubin’s rules [20, 21].

## Results

### Characteristics of study subjects

In total, 1,111,809 EMS records were collected between January 2015 and December 2017, of which 483,460 patients were transported from the scene of injury to a trauma center with A1 or A2 priority. After exclusion of non-trauma patients (*n* = 369,001), 114,459 patients were included (Fig. 1). The patients included in this cohort had a median age of 58.2 years, 56,798 (49.6%) were female and the dispatch center assigned 52,238 (45.6%) patients with A1 priority (Table 2). Ambulances dispatched with A1 and A2 priority had a median response time of 7.5 min and 11.0 min (*p* < 0.001), respectively. The 35,974 (31.4%) patients that were hospitalized had a mean ISS of 7.5 ± 6.4. In 20,285 (17.7%) patients, there were differences between dispatch and transport priorities (Table 3). Patients with a lowered priority (i.e., A1 dispatch priority lowered to A2 transport priority) more frequently had stable vital signs (*p* < 0.001), longer transport times (*p* < 0.001), fewer injuries to head or neck (*p* < 0.001), thorax (*p* < 0.001), and abdomen (*p* < 0.001), and had less critical-resource use (*p* < 0.001) than patients with a maintained A1 transport priority. Conversely, patients with a raised priority (i.e., A2 dispatch priority raised to A1 transport priority) more often had hypotension (*p* < 0.001), abnormal respiratory rates (*p* < 0.001), or an impaired Glasgow Coma Scale (*p* < 0.001) compared to patients with a maintained A2 transport priority. Their median transport time was comparable (0.1 min shorter, *p* = 0.725) and their median driving distance was 2.3 km longer (*p* < 0.001). Head or neck (*p* < 0.001), thoracic (*p* < 0.001), and abdominal injuries (*p* < 0.001) were more prevalent, while fewer injuries of their extremities (*p* < 0.001) were seen. They required five times more critical resources (*p* < 0.001).

**Table 2:** Cohort characteristics

	All patients (*n* = 114,459)	A1 dispatch priority (*n* = 52,238)	A2 dispatch priority (*n* = 62,221)	*p* value
Demographic characteristics				
Age	58.2 (30.8–78.4)	48.5 (25.5–68.6)	68.3 (40.7–82.9)	< 0.001
Female gender	56,798 (49.6)	21,441 (41.0)	35,357 (56.8)	< 0.001
Outcomes				
Ambulance response time (min)	9.1 (6.3–12.8)	7.5 (5.3–10.1)	11.0 (7.8–14.9)	< 0.001
Initial destination: higher-level TC	24,918 (21.8)	14,284 (27.3)	10,634 (17.1)	< 0.001
In-hospital stay	35,974 (31.4)	16,165 (30.9)	19,809 (31.8)	0.001
ISS^*^, mean (SD)	7.5 (6.4)	8.2 (8.5)	7.0 (4.0)	< 0.001
Mortality	1606 (1.4)	737 (1.4)	869 (1.4)	0.858
Undertriage rate, % (95% CI)				
Early critical-resource use	30.3 (28.8–31.9)	24.4 (22.9–36.0)	60.6 (56.5–64.7)	
ISS ≥ 16	25.5 (24.0–27.1)	19.8 (18.2–21.4)	52.5 (48.2–56.9)	
Overtriage rate, % (95% CI)				
Early critical-resource use	20.3 (20.1–20.6)	24.6 (24.2–25.0)	16.9 (16.6–17.2)	
ISS ≥ 16	20.4 (20.2–20.7)	24.9 (24.5–25.2)	16.8 (16.6–17.1)	

Data are median (IQR) or *n* (%), unless otherwise stated. Variables with missing data were dispatch center priority (0.1%) and response time (1.4%). These variables were multiply imputed and rounded

*TC* Trauma Center, *ISS* Injury Severity Score, *SD* Standard Deviation, *min* minutes, *CI* Confidence Interval

*Hospitalized patients;

**Table 3:** Outcomes per priority subgroup

	A1 dispatch priority (*n* = 52,238)			A2 dispatch priority (*n *= 62,221)		
	A1 transport priority (*n* = 34,613)	A2 transport priority (*n* = 17,625)	*p* value^a^	A1 transport priority (*n* = 2660)	A2 transport priority (*n* = 59,561)	*p* value^b^
Demographics						
Age	48.1 (25.4–68.3)	49.1 (25.8–69.0)	0.003	57.9 (30.8–78.0)	68.7 (41.3–83.0)	< 0.001
Female gender	13,889 (40.1)	7552 (42.8)	< 0.001	1317 (49.5)	34,041 (57.2)	< 0.001
Prehospital vital signs						
Systolic blood pressure < 90 mmHg	766 (2.2)	275 (1.6)	< 0.001	65 (2.4)	640 (1.1)	< 0.001
Respiratory rate > 29 or < 10	1505 (4.3)	485 (2.8)	< 0.001	78 (2.9)	982 (1.6)	< 0.001
Glasgow Coma Scale score < 14	3833 (11.1)	1129 (6.4)	< 0.001	144 (5.4)	1557 (2.6)	< 0.001
Outcomes						
Ambulance response time (min)	7.4 (5.2–10.1)	7.6 (5.4–10.0)	0.001	10.7 (7.3–14.2)	11.0 (7.8–15.0)	< 0.001
Ambulance transport time (min)	12.1 (7.6–18.2)	12.6 (8.0–18.0)	< 0.001	13.0 (8.7–18.2)	13.1 (8.3–18.6)	0.725
Distance to destination (km)	9.1 (4.3–17.1)	9.3 (4.4–16.0)	0.097	11.3 (5.3–17.5)	9.0 (4.3–15.7)	< 0.001
Initial destination: higher-level TC	10,386 (29.1)	3898 (21.0)	< 0.001	715 (20.5)	9919 (16.9)	< 0.001
In-hospital stay	11,484 (33.2)	4682 (26.6)	< 0.001	953 (35.8)	18,856 (31.7)	< 0.001
Severe injury (AIS score ≥ 3)						
Head and neck	1954 (5.6)	537 (3.0)	< 0.001	85 (3.2)	600 (1.0)	< 0.001
Face	56 (0.2)	9 (0.1)	0.002	2 (0.1)	4 (0.0)	0.024^†^
Thorax	1398 (4.0)	334 (1.9)	< 0.001	73 (2.7)	505 (0.8)	< 0.001
Abdomen	354 (1.0)	66 (0.4)	< 0.001	15 (0.6)	88 (0.1)	< 0.001
Extremities	1483 (4.3)	659 (3.7)	0.004	287 (10.8)	9210 (15.5)	< 0.001
ISS*, mean (SD)	8.8 (9.1)	6.7 (6.3)	< 0.001	7.6 (5.9)	6.9 (3.9)	< 0.001
Early critical-resource use*	2385 (6.9)	403 (2.3)	< 0.001	94 (3.5)	445 (0.7)	< 0.001
ISS ≥ 16*	1955 (5.6)	397 (2.3)	< 0.001	85 (3.2)	416 (0.7)	< 0.001
Mortality	598 (1.7)	139 (0.8)	< 0.001	37 (1.4)	832 (1.4)	0.812
Undertriage rate (95%CI)						
Early critical-resource use	22.7 (21.1–24.4)	34.5 (29.9–39.3)		37.6 (28.3–47.8)	65.5 (61.0–69.8)	
ISS ≥ 16	16.7 (15.1–18.4)	34.8 (30.2–39.7)		37.2 (27.4–48.3)	55.7 (50.8–60.4)	
Overtriage rate (95%CI)						
Early critical-resource use	26.5 (26.0–27.0)	21.1 (20.5–21.7)		25.6 (23.8–27.4)	16.5 (16.2–16.8)	
ISS ≥ 16	26.8 (26.3–27.3)	21.1 (20.5–21.7)		25.7 (23.9–27.6)	16.5 (16.1–16.8)	

Data are median (IQR) or *n* (%), unless otherwise stated. Variables with missing data were dispatch priority (0.1%), transport priority (3.0%), systolic blood pressure (29.1%), respiratory rate (36.6%), Glasgow Coma Scale (13.6%), response time (1.4%), and transport time (4.1%). These variables were multiply imputed and rounded

*TC* Trauma Center, *AIS* Abbreviated Injury Scale, *ISS* Injury Severity Score, *SD* Standard Deviation, *min* minute, *km* kilometer, *CI* Confidence Interval

^a^A1 vs A2 transport priority in A1 dispatch priority group

^b^A1 vs. A2 transport priority in A2 dispatch priority group

*Hospitalized patients

^†^Fisher’s exact test

### Main outcomes

**Fig. 2:**
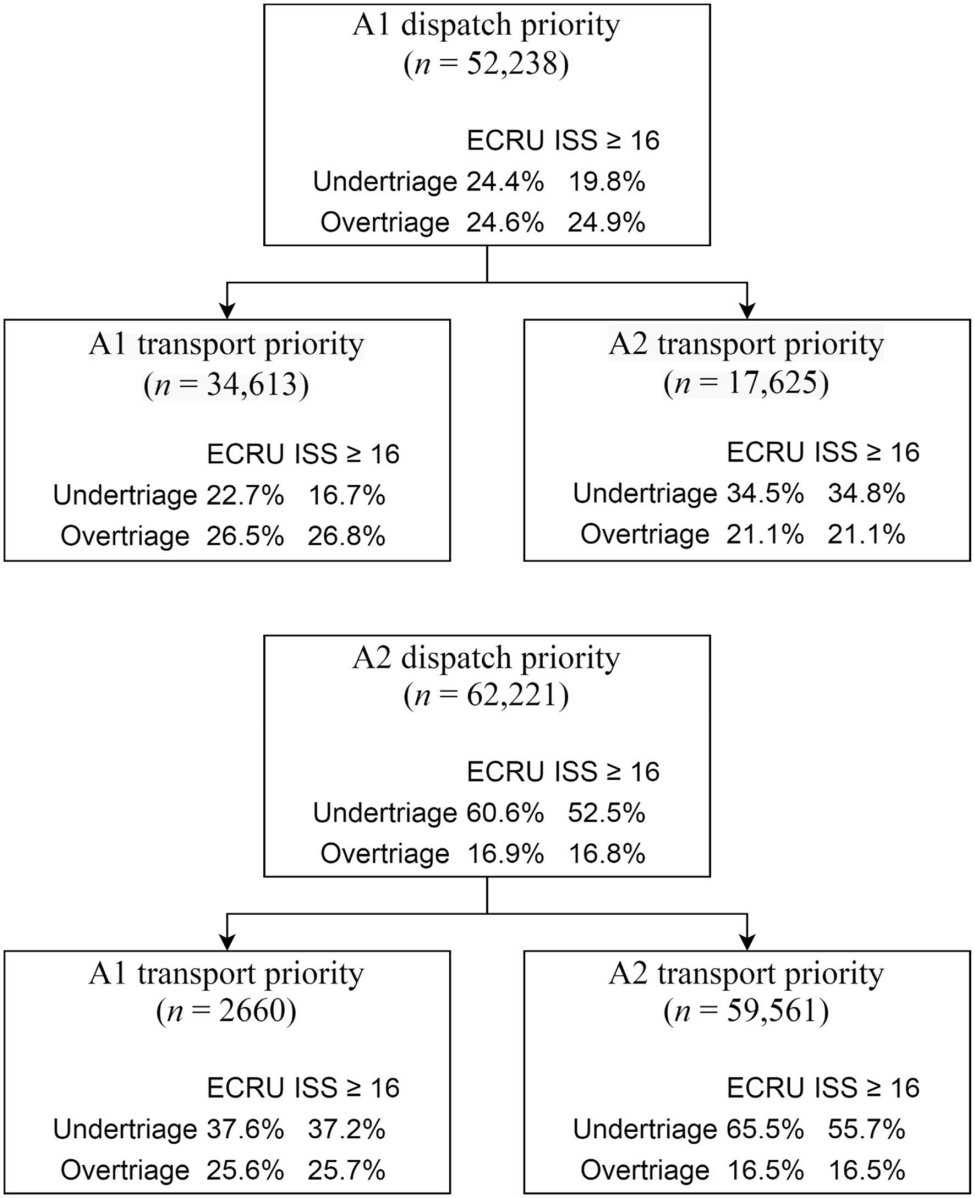
Triage rates per priority subgroup according to both reference standards. *ECRU* Early critical-resource use; *ISS* Injury Severity Score

The dispatch center has set A1 priority in 83.8% (82.5–85.0) of the patients with early critical-resource use (Table 4). The dispatch priority sensitivity in patients with an ISS ≥ 16 was 82.5% (81.0–83.8). The transport priority had a sensitivity of 74.5% (73.0–76.0) and 71.5% (69.8–73.2) according to both reference standards. Figure 2 illustrates the triage rates in the different priority subgroups according to the primary reference standard. The undertriage rate in patients with A1 dispatch and transport priority based on early critical-resource use was 22.7% (95% CI, 21.1–24.4). The 17,625 (15.4%) patients of whom the priority was lowered by the EMS professional had an undertriage rate of 34.5% (29.9–39.3). Evaluation of these priorities using the ISS resulted in undertriage rates ranging from 16.7 (15.1–18.4) to 34.8% (30.2–39.7). Otherwise, 65.5% (61.0–69.8) of the patients in need of specialized care with A2 dispatch and transport priority were transported to lower-level trauma centers. The undertriage rate of these patients decreased to 37.6% (28.3–47.8) when their transport priority was raised. According to the secondary reference standard (ISS ≥ 16), a raised priority by the EMS professional was associated with a decrease in undertriage from 55.7% (50.8–60.4) to 37.2% (27.4–48.3).

**Table 4:** Priority sensitivity by professionals at dispatch centers and EMS

	Dispatch center	EMS professional
Early critical-resource use, sensitivity (95% CI)	83.8 (82.5–85.0)	74.5 (73.0–76.0)
ISS ≥ 16, sensitivity (95%-CI)	82.5 (81.0–83.8)	71.5 (69.8–73.2)

*EMS* Emergency Medical Services, *CI* Confidence Interval, *ISS* Injury Severity Score;

## Discussion

This cohort study evaluated the accuracy of dispatch center and EMS professional prioritization of trauma patients in need of specialized care. We have reported that the dispatch center assigned 83.8% of these patients with the highest priority. 74.5% of the patients requiring specialized care were transported to a trauma center with A1 priority. Moreover, we found that undertriage rates varied widely between patients with A1 priority (22.7%) and patients with A2 priority (65.5%). There was less variety in overtriage rates, ranging from 26.5% to 16.5%. Differences between dispatch and transport priority were present in 17.7% of the patients. In 15.4% of the cases, the priority was lowered, while the priority was raised in 2.3%. Our results showed that an A1 dispatch priority was associated with shorter response times. Patients with a lowered transport priority demonstrated an increased transport time and distance to destination. Conversely, patients with a raised transport priority had comparable transport times but their median distance to destination was longer. There were geographic differences between the EMS regions which complicate the interpretation of these results (Supplementary Table 1). Also, a counter-intuitive finding was that patients assigned with A1 dispatch priority had a slightly lower mortality in comparison to patients assigned with A2 priority. This is likely due to a large difference in age (median 48.9 vs. 68.4).

A major strength of this study is our broad inclusion criteria—including all trauma patients transported by the participating EMS regions—and highly accurate patient selection procedure that minimized selection bias [11]. Trauma patients were included from eight EMS regions that cover urban, suburban and rural areas. These regions also differed in protocols and training programs. The diversity of these EMS regions contributes to the generalizability of our results. Furthermore, data were prospectively collected in a systematic matter by all EMS regions and trauma centers. All trauma centers within the participating trauma regions contributed to data collection.

In the current study, we chose to evaluate priority-setting accuracy in terms of sensitivity. Because patients in need of specialized care benefit from swift transportation to a higher-level trauma center, we assumed that being assigned with A1 priority would favor their outcomes. On the other hand, it can be reasoned that patients without the need for specialized care also benefit from the highest dispatch and transport priority. Therefore, we argue that it would be invalid to report specificity and diagnostic predictive values. The distance to destination of patients that had their priority raised by the EMS professional was longer in comparison to patients for which the high priority was maintained. This finding suggests that the transport priority might be affected by the driving distance. Future research should investigate whether a causal relation exists between priority-setting and distance to trauma centers.

Our findings are in line with two studies that demonstrated a dispatch priority sensitivity of 82.6% [22] and 86% [23]. However, these studies also included patients suffering from stroke and cardiac complaints. To the best of our knowledge, the current study is the first to investigate priority-setting by dispatch centers and EMS professionals in a population consisting of exclusively trauma patients. A previous investigation reported that in 73% of the patients with the highest dispatch center priority, the priority was lowered by the EMS professionals [24]. The authors suggest that this may have been caused by a safety margin in the assessment of the operators at the dispatch center. Additionally, they reported a raised priority in 3.5% of the patients with the second-highest dispatch center priority, in accordance with our findings (2.8%).

Dispatch centers in our current cohort used different dispatch protocols with a comparable priority sensitivity. Two regions used a Criteria-Based Dispatch Protocol (81.8%), whereas the other three dispatch centers operated on a Medical Priority Dispatch System (84.9%). Both systems are used widely across modern trauma systems and there is no consensus on which system or protocol is superior [25]. Moreover, Bohm and Kurland [7] conducted a systematic review and concluded that there was little evidence describing the accuracy of any medical dispatch system.

Early critical-resource use was used as the primary reference standard. Although the ISS has been recommended by the American College of Surgeons to evaluate triage accuracy of trauma systems, it has been suggested that a resource-based standard is a better alternative to determine whether a patient is in need of specialized care or not [15, 17, 26]. Our reference standard differs from previously suggested resource-based endpoints, as the administration of blood products was not available in our dataset [14, 16]. A limitation of this composite endpoint is that some resources (e.g., neurosurgical care) are unavailable in lower-level trauma centers. Also, the lower volume of patients in need of specialized care in these centers affects the use of and experience with certain resources. However, our results show that there is little difference in terms of sensitivity, under- and overtriage for both reference standards.

Future research should focus on the predictive value of dispatch center prioritization. The addition of dispatch priority to existing prediction models could improve the identification of patients in need of specialized care by EMS professionals during field triage. Furthermore, to achieve optimal resource use and patient outcomes, effort should be put into the investigation of highly accurate priority criteria.

## Limitations

The current study suffers from some limitations. First, the amount of missing data in some variables, such as systolic blood pressure (29.1%) and respiratory rate (36.6%), was considerable. However, the determinants dispatch and transport priority were only missing in a minority of the cases. We performed multilevel multiple imputation accounting for clustering to impute these missing values and to create an actual representation. Also, we calculated the driving distance from the scene of injury to the trauma center with Bing Maps [12]. This navigation software has been developed for regular traffic. Because ambulances are allowed to use alternate routes, we may have overestimated the driving distance. Furthermore, some patients were treated on-scene and were not transported to a trauma center. However, the exclusion of these patients will probably not have affected our results as we assume that the vast majority of these patients were not in need of specialized care. Also, low-priority transports were excluded from this study, because these are scheduled transports. We assume that these low-priority cases do not involve patients in need of specialized care and will therefore not have affected our main outcomes. Finally, we conducted this study in nurse-staffed EMS regions, thereby limiting the generalizability of our results to regions with ambulances staffed by physicians.

## Conclusion

In conclusion, this study has evaluated and compared the accuracy of dispatch and transport priority. Professionals at the dispatch centers and EMS assigned the majority of the patients in need of specialized care with the highest priority. To improve the outcome of patients requiring specialized care and to achieve the most efficient use of resources, future research should investigate the effectiveness of highly accurate priority criteria.

## Supplementary Information

Below is the link to the electronic supplementary material.

Supplementary file1 (DOCX 19 kb)

## Data Availability

Available upon reasonable request, approval of the participating EMS and trauma regions, and provided that appropriate ethical approval is sought and approved.

## Code availability

Available upon request.

## References

[1] MacKenzie EJ, Rivara FP, Jurkovich GJ, Nathens AB, Frey KP, Egleston BL (2006). A national evaluation of the effect of trauma-center care on mortality. N Engl J Med.

[2] Staudenmayer K, Weiser TG, Maggio PM, Spain DA, Hsia RY (2016). Trauma center care is associated with reduced readmissions after injury. J Trauma Acute Care Surg.

[3] Polites SF, Leonard JM, Glasgow AE, Zielinski MD, Jenkins DH, Habermann EB (2018). Undertriage after severe injury among United States trauma centers and the impact on mortality. Am J Surg.

[4] Cudnik MT, Newgard CD, Sayre MR, Steinberg SM (2009). Level I versus level ii trauma centers: an outcomes-based assessment. J Trauma Injury Infect Critical Care.

[5] Newgard CD, Staudenmayer K, Hsia RY, Mann NC, Bulger EM, Holmes JF (2013). The cost of overtriage: more than one-third of low-risk injured patients were taken to major trauma centers. Health Aff (Millwood).

[6] Harmsen AM, Giannakopoulos GF, Moerbeek PR, Jansma EP, Bonjer HJ, Bloemers FW (2015). The influence of prehospital time on trauma patients outcome: a systematic review. Injury.

[7] Bohm K, Kurland L (2018). The accuracy of medical dispatch—a systematic review. Scand J Trauma Resusc Emerg Med.

[8] Bos N, Krol M, Veenvliet C, Plass AM. Ambulance care in Europe: organization and practices of ambulance services in 14 European countries. NIVEL, Utrecht. 2015. https://www.nivel.nl/sites/default/files/bestanden/Rapport_ambulance_care_europe.pdf. Accessed 18 Jan 2021.

[9] Rolink M, Bos N, de Boer D. Urgentie in de ambulancezorg en de acute eerstelijns zorgketen: Een verantwoording voor de urgentie-indeling. NIVEL, Utrecht. 2019. https://nivel.nl/sites/default/files/bestanden/Rapport_urgentie_ambulancezorg.pdf. Accessed 18 Jan 2021.

[10] von Elm E, Altman DG, Egger M, Pocock SJ, Gøtzsche PC, Vandenbroucke JP (2007). The Strengthening the Reporting of Observational Studies in Epidemiology (STROBE) statement: guidelines for reporting observational studies. Lancet.

[11] van der Sluijs R, Lokerman RD, Waalwijk JF, de Jongh MAC, Edwards MJR, den Hartog D (2020). Accuracy of pre-hospital trauma triage and field triage decision rules in children (P2–T2 study): an observational study. Lancet Child Adolesc Health.

[12] Bing Maps. Microsoft Cooperation, Redmond. 2021. https://www.bing.com/maps. Accessed 19 Jan 2021.

[13] Voskens FJ, van Rein EAJ, van der Sluijs R, Houwert RM, Lichtveld RA, Verleisdonk EJ (2018). Accuracy of prehospital triage in selecting severely injured trauma patients. JAMA Surg.

[14] Lerner EB, Cushman JT, Drendel AL, Badawy M, Shah MN, Guse CE (2017). Effect of the 2011 revisions to the field triage guidelines on under- and over-triage rates for pediatric trauma patients. Prehosp Emerg Care.

[15] Lerner EB, Willenbring BD, Pirrallo RG, Brasel KJ, Cady CE, Colella MR (2014). A consensus-based criterion standard for trauma center need. J Trauma Acute Care Surg.

[16] Newgard CD, Fu R, Zive D, Rea T, Malveau S, Daya M (2016). Prospective Validation of the National Field Triage Guidelines for Identifying Seriously Injured Persons. J Am Coll Surg.

[17] American College of Surgeon Committee on Trauma. Resources for Optimal Care of the Injured Patient. Chicago, IL; 2014. https://www.facs.org/quality-programs/trauma/tqp/center-programs/vrc/resources. Accessed 19 Jan 2021.

[18] R Development Core Team. R: A Language and Environment for Statistical Computing. Vienna, Austria: R Foundation for Statistical Computing; 2021. https://www.R-project.org/

[19] Audigier V, Resche-Rigon M. Micemd: Multiple Imputation by Chained Equations with Multilevel Data. Version 1.6.0; 2019. https://rdrr.io/cran/micemd/man/micemd-package.html. Accessed 18 Jan 2021.

[20] Rubin DB (1987). Multiple imputations for nonresponse in surveys.

[21] Agresti A, Coull BA (1998). Approximate is better than “exact” for interval estimation of binomial proportions. Am Stat.

[22] Torlen K, Kurland L, Castren M, Olanders K, Bohm K (2017). A comparison of two emergency medical dispatch protocols with respect to accuracy. Scand J Trauma Resusc Emerg Med.

[23] Dami F, Golay C, Pasquier M, Fuchs V, Carron PN, Hugli O (2015). Prehospital triage accuracy in a criteria based dispatch centre. BMC Emerg Med.

[24] Khorram-Manesh A, LennquistMontan K, Hedelin A, Kihlgren M, Ortenwall P (2011). Prehospital triage, discrepancy in priority-setting between emergency medical dispatch centre and ambulance crews. Eur J Trauma Emerg Surg.

[25] Lyon RM, Bohm K, Christensen EF, Olasveengen TM, Castren M (2013). The inaugural European emergency medical dispatch conference—a synopsis of proceedings. Scand J Trauma Resusc Emerg Med.

[26] Newgard CD, Hedges JR, Diggs B, Mullins RJ (2008). Establishing the need for trauma center care: anatomic injury or resource use?. Prehosp Emerg Care.

