# Supplementary material for: Priority accuracy by dispatch centers and Emergency Medical Services professionals in trauma patients: a cohort study
Source: Eur J Trauma Emerg Surg. 2021 May 21;48(2):1111–20. doi: 10.1007/s00068-021-01685-1 (PMC9001562; doi:10.1007/s00068-021-01685-1)
Supplement: Supplementary file 1 — Supplementary file1 (DOCX 19 kb) [file 68_2021_1685_MOESM1_ESM.docx]

**Supplementary content**Supplement to: Waalwijk JF, Lokerman RD, van der Sluijs R, et al. Priority accuracy by dispatch centers and Emergency Medical Services professionals in trauma patients: a cohort study.

**Supplementary Table 1.** Geographic characteristics per EMS region.

| **Supplementary Table 1. Geographic characteristics per EMS region** | | | | | | |  |
| --- | --- | --- | --- | --- | --- | --- | --- |
|  | **Brabant Midden-West** (n=30,194) | **Brabant-Noord** (n=15,682) | **Gelderland-Zuid** (n=11,949) | **Rotterdam-Rijnmond** (n=34,919) | **Utrecht** (n=21,715) | | |
| **Dispatch priority A1 and transport priority A1** | | | | | |  |  |
| Patients | 7440 (24.7) | 4465 (28.5) | 3745 (31.3) | 15,934 (45.6) | 3030 (14.0) | |  |
| Response time (min) | 7.9 (5.8-10.5) | 8.2 (5.8-10.8) | 8.1( 5.7-11.0) | 6.8 (4.8-9.4) | 7.0 (5.0-10.0) | |  |
| Transport time (min) | 13.9 (9.1-19.7) | 13.8 (9.4-18.6) | 14.0 (8.2-20.0) | 10.4 (6.5-16.7) | 12.0 (8.0-18.0) | |  |
| Distance to destination (km) | 13.6 (6.5-20.7) | 13.0 (7.1-18.0) | 12.2 (4.8-21.0) | 5.6 (3.3-11.4) | 13.8 (7.5-20.8) | |  |
| Transport speed (km/h), mean (SD) | 64.1 (56.0) | 56.8 (38.3) | 57.5 (51.0) | 42.1 (34.9) | 77.4 (58.8) | |  |
| **Dispatch priority A1 and transport priority A2** | | | | | | |  |
| Patients | 5772 (19.1) | 2538 (16.2) | 717 (6.0) | 3785 (10.8) | 4813 (22.2) | |  |
| Response time (min) | 7.7 (5.7-10.2) | 7.9 (5.6-10.6) | 8.5 (6.0-11.5) | 7.1 (4.9-9.7) | 7.0 (5.0-10.0) | |  |
| Transport time (min) | 13.4 (8.8-18.8) | 13.6 (9.3-18.0) | 14.9 (8.9-20.5) | 10.3 (6.7-16.8) | 12.0 (8.0-17.0) | |  |
| Distance to destination (km) | 12.2 (5.4-18.1) | 12.4 (6.5-16.7) | 13.0 (4.7-20.3) | 5.3 (3.1-9.7) | 8.5 (4.4-14.6) | |  |
| Transport speed (km/h), mean (SD) | 59.2 (51.2) | 55.2 (41.6) | 53.5 (38.5) | 38.2 (30.8) | 50.2 (43.3) | |  |
| **Dispatch priority A2 and transport priority A1** | | | | | | |  |
| Patients | 730 (2.4) | 375 (2.4) | 171 (1.4) | 623 (1.8) | 760 (3.5) | |  |
| Response time (min) | 10.8 (8.0-14.4) | 11.7 (8.4-15.1) | 9.1 (6.5-13.2) | 8.8 (5.8-12.6) | 12.0 (8.0-16.0) | |  |
| Transport time (min) | 14.7 (10.0-19.8) | 13.8 (9.9-17.8) | 14.0 (7.9-18.9) | 11.5 (7.3-17.5) | 12.0 (8.0-17.0) | |  |
| Distance to destination (km) | 13.0 (6.2-18.7) | 12.7 (7.4-16.6) | 9.1 (4.0-17.3) | 6.3 (3.5-13.1) | 12.8 (7.1-19.1) | |  |
| Transport speed (km/h), mean (SD) | 60.2 (53.8) | 55.8 (44.3) | 50.8 (47.3) | 41.7 (28.5) | 70.7 (63.8) | |  |
| **Dispatch priority A2 and transport priority A2** | | | | | |  |  |
| Patients | 16,252 (53.8) | 8304 (53.0) | 7316 (61.2) | 14,577 (41.7) | 13,112 (60.4) | |  |
| Response time (min) | 10.8 (8.0-14.4) | 11.1 (8.0-14.7) | 11.1 (7.6-14.9) | 10.0 (7.0-13.9) | 12.0 (8.0-16.0) | |  |
| Transport time (min) | 14.3 (9.4-19.5) | 13.8 (9.6-17.8) | 14.0 (8.3-19.5) | 10.8 (6.9-17.1) | 13.0 (9.0-19.0) | |  |
| Distance to destination (km) | 12.5 (5.7-17.9) | 11.8 (6.4-16.0) | 9.7 (4.0-17.7) | 5.4 (3.2 -10.3) | 8.3 (4.5-13.9) | |  |
| Transport speed (km/h), mean (SD) | 57.7 (51.7) | 51.5 (36.1) | 48.5 (46.6) | 38.2 (32.4) | 45.4 (39.7) | |  |
| EMS: Emergency Medical Services; min: minutes; km: kilometer; km/h: kilometers per hour; SD: Standard Deviation;  Data are median (IQR) or n (%), unless otherwise stated. Variables with missing data were dispatch center priority (0.1%), transport priority (3.0%), response time (1.4%), and transport time (4.1%). These variables were multiply imputed and rounded. | | | | | | |  |
